# Identification of Key Residues for Urate Specific Transport in Human Glucose Transporter 9 (hSLC2A9)

**DOI:** 10.1038/srep41167

**Published:** 2017-01-24

**Authors:** Wentong Long, Rashmi Panigrahi, Pankaj Panwar, Kenneth Wong, Debbie O′Neill, Xing-Zhen Chen, M. Joanne Lemieux, Chris I. Cheeseman

**Affiliations:** 1 Department of Physiology, Faculty of Medicine and Dentistry, University of Alberta, Alberta, Canada; 2 Department of Biochemistry, Faculty of Medicine and Dentistry, University of Alberta, Alberta, Canada

## Abstract

Human glucose transporter 9 (hSLC2A9) is critical in human urate homeostasis, for which very small deviations can lead to chronic or acute metabolic disorders. Human SLC2A9 is unique in that it transports hexoses as well as the organic anion, urate. This ability is in contrast to other homologous sugar transporters such as glucose transporters 1 and 5 (SLC2A1 & SLC2A5) and the xylose transporter (XylE), despite the fact that these transporters have similar protein structures. Our *in silico* substrate docking study has revealed that urate and fructose bind within the same binding pocket in hSLC2A9, yet with distinct orientations, and allowed us to identify novel residues for urate binding. Our functional studies confirmed that N429 is a key residue for both urate binding and transport. We have shown that cysteine residues, C181, C301 and C459 in hSLC2A9 are also essential elements for mediating urate transport. Additional data from chimæric protein analysis illustrated that transmembrane helix 7 of hSLC2A9 is necessary for urate transport but not sufficient to allow urate transport to be induced in glucose transporter 5 (hSLC2A5). These data indicate that urate transport in hSLC2A9 involves several structural elements rather than just a unique substrate binding pocket.

For over half a century, structure-function studies have primarily focused on the identification of single substrate binding pockets within the aqueous channels of transporter proteins^1^,^2^,^3^. However, recent kinetic and substrate binding studies of glucose transporters reveal that a single binding pocket may not fully explain how a transporter protein selects and translocates its substrates. In addition to key amino acid residues forming a potential binding pocket, other structural features such as interactions between transmembrane helices, intracellular helices, disulfide bridges, and salt bridges have been implicated in governing substrate specificity in transporter function^4^,^5^,^6^,^7^.

Within the Class II human glucose transporters (hSLC2As), hSLC2A9 and hSLC2A5 have the highest sequence homology, with 29% identity and 59% similarity^8^,^9^, yet they appear to interact with substrates very differently. Human SLC2A5 is primarily a high affinity fructose transporter, whereas, hSLC2A9 is a very high affinity fructose and a high capacity urate transporter. This difference between the two transporters raises a question as to how their structural differences allow them to transport different substrates. The recently resolved glucose transporter 5 (SLC2A5) crystal structure reveals that its substrate binding pocket includes residues Tyr31, Gln287, Gln288, His386, His418, and Trp419^7^. In addition, most of these residues are conserved in the fructose binding pocket in our hSLC2A9 model based on this SLC2A5 crystal, except Gln287 (Tyr298 in hSLG2A9) and His418 (Asn429) (Fig. 1). We reasoned that these two residues might be critical for hSLC2A9 to handle urate and fructose transport.

Our earlier studies have revealed that a single hydrophobic residue in transmembrane helix 7 (H7), is a major determinant of substrate selectivity in the hGLUTs^10^,^11^. Substitution of isoleucine into valine in the fructose transporting hSLC2As only affects fructose but not glucose transport. This hydrophobic interaction also appears to have no effect on urate transport in hSLC2A9^10^,^12^. These findings also support the hypothesis that hexoses and urate have different binding residues in hSLC2A9. Moreover, multi-amino acid sequence alignments of Class I and II hSLC2As revealed that hSLC2A9 has very distinct cysteine residues compared to other SLC2As (Suppl. Fig. 1). Earlier studies have also indicated that cysteine residues might be critical for hexose translocation in hSLC2A1^13^,^14^,^15^,^16^. Nevertheless, the importance of cysteine residues in urate transport in hSLC2A9 has not been previously examined.

In this study, we examined the basis for the distinct substrate transport abilities of the hSLC2A9 transporter. Our SLC2A9 molecular docking studies based on the newly reported SLC2A5 crystal structure suggest that urate binds within the same translocation pocket as fructose; however, the two substrates interact with some distinct residues. Furthermore, the docking analysis reveals that the binding orientation of urate differs significantly from fructose in hSLC2A9. Our results from radiolabelled flux studies and electrophysiological studies confirm these findings. In addition, we identified hydrophobic residues between transmembrane helices that affect fructose but not urate transport, and *vice versa* using hSLC2A9/hSLC2A5 chimeras. we examined the role of all cysteine residues in HSLC2A9. Finally, This study provides strong evidence that urate and fructose transport mediated by hSLC2A9 involves multiple structural elements that together provide a unique ability to handle these two very different substrates.

## Results

### Comparative urate/fructose docking analysis and mutagenesis studies of the possible urate binding-site residues in hSLC2A9

Both SLC2A9 and SLC2A5 can transport fructose, yet only SLC2A9 can transport urate. Notably, crucial residues involved in fructose binding have been investigated by crystal structure guided alanine substitution mutations^7^. Although most of these residues are conserved in SLC2A9 and SLC2A5, sequence alignment reveals residues that may play important roles in the distinct urate transport abilities of SLC2A9. We questioned whether fructose/urate binding sites are conserved or different between SLC2A9 and SLC2A5. If their binding sites are very similar, do the non-conserved residues within the putative binding pocket play a specific role in urate binding, or does urate bind to distinct sites that differ from the ones for fructose ? To address these questions and identify key residues, we performed *in silico* docking studies to examine both fructose and urate binding sites utilizing the molecular modeling of hSLC2A9b based on the SLC2A5 crystal structure. hSLC2A9b has an identical amino acid sequence to the full-length hSLC2A9, yet has a shorter N-terminus that is predicted to reside outside of the membrane and not influence transport, but instead targets the two isoforms to different membrane domains within epithelial cells^17^. The use of hSLC2A9b allowed us to have a refined molecular model compared to the full length hSLC2A9 whose N-terminus was disordered during structure prediction. Importantly, both isoforms have similar urate and fructose transport activities^17^,^18^.

**Figure 1:**
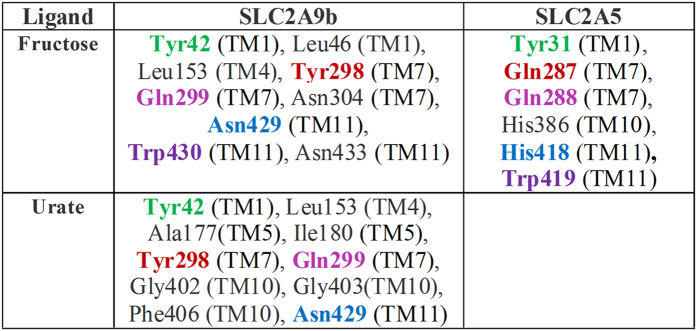
List of residues involved in interaction with urate in SLC2A9 and fructose in SLC2A9/SLC2A5. Note: The residues of SLC2A9 and SLC2A5 located at the similar position are color coded. The transmembrane helices to which the residues belong have been noted.

**Figure 2:**
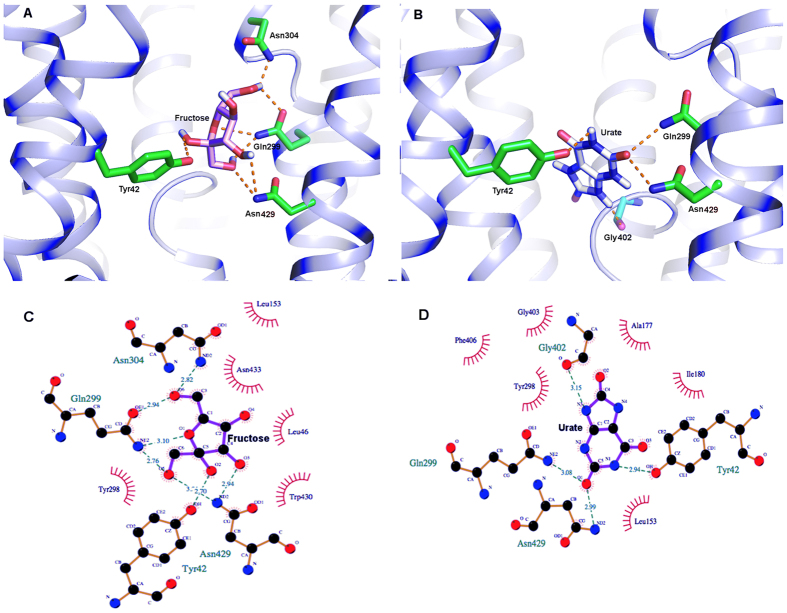
Docking studies of human SLC2A9b with fructose and urate. Panel (A and B) Binding pocket for fructose and urate, respectively. The residues involved in hydrogen bonding interaction (orange dash lines) have been shown in stick (green). The nitrogen and oxygen atoms are shown in blue and red respectively. The nitrogen and oxygen atoms are shown in blue and red respectively. Panel (C and D) Ligplot showing all the residues forming the binding pocket for fructose and urate, respectively. Hydrogen bonding interactions are shown in green dashed lines and the hydrophobically interacting pairs of atom are shown in red lines patterns.

Our *in silico* docking analysis revealed that both fructose and urate bind to the same binding pocket in hSLC2A9b, which is indeed similar to that identified for the fructose transporter, SLC2A5^7^ (Fig. 1). The two ligands have different orientations within the binding pocket. In hSLC2A9b, fructose docks in a plane parallel to the pore axis of the transporter. In contrast, urate is in a plane almost perpendicular to the axis through the pore (Fig. 2A and B). We identified three residues from the N-terminal transmembrane helices (H1-H6), and six residues from the C-terminal transmembrane helices (H7-H12) that could be involved in substrate binding (Figs 1 and 2). This asymmetric substrate binding between the two six transmembrane domains has also been reported in other sugar transporters such as SLC2A5, SLC2A1 and XylE^4^,^5^,^7^. Of these residues, some are conserved in the substrate binding pocket, namely Y42 and W430, (Fig. 1). In SLC2A5, the equivalent W419 has altered intrinsic fluorescence in the presence of fructose^7^. Importantly, substrate binding residues in SLC2A5 such as N287 and H418 have been replaced by residues Y298 and N429 in the urate transporter SLC2A9, suggesting these residues may be involved in urate binding.

**Figure 3:**
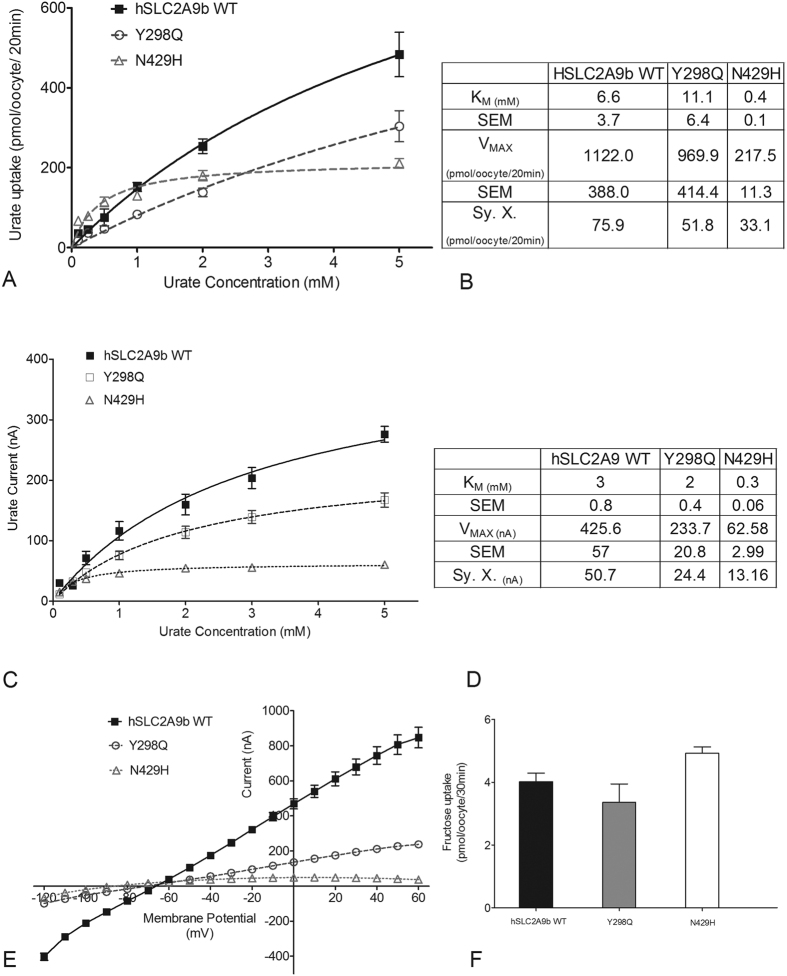
Urate and fructose transport mediated by WT hSLC2A9 and its Y298Q and N429H mutants. Panel (**A**) Michaelis-Menten curves of ^14^C urate kinetics of hSLC2A9 WT (▪), Y298Q (○) and N429H (Δ). Panel (**B**) ^14^C urate kinetic constants and the standard error of the regression (Sy. X) of the three isoforms (n ≥ 3). Panel (**C**) Michaelis-Menten curves of urate-induced currents of WT hSLC2A9, Y298Q and N429H mutants. Panel (**D**) Urate-induced current kinetic constants and the standard error of the regression (Sy. X) of the WT and two mutants (n ≥ 15 oocytes from 3 frogs). Panel (**E**) Current-voltage curve of 1 mM urate-induced current obtained using a RAMP protocol WT hSLC2A9 (▪), Y298Q (○) and N429H (Δ) mutant expressing oocytes. (n ≥ 15 oocytes from 3 frogs). Panel (**F**) ^14^C fructose uptake mediated by WT hSLC2A9 and its mutants. (n ≥ 3, One-way ANOVA, *p < 0.05).

To assess whether these distinct hSLC2A9b residues, Y298 and N429, in the substrate binding pocket account for urate transport, they were mutated into the corresponding residues in the fructose transporter hSLC2A5, Y298Q and N429H. All mutations in SLC2A9b were assessed for expression in oocytes (Suppl. Fig. 2). Both ^14^C urate flux and TEVC studies indicate that the Y298Q mutant transports urate similarly to hSLC2A9b, whereas N429H has lower V_MAX_ and much lower K_M_ values compared to WT hSLC2A9b (Fig. 3A–D). Additionally, we examined urate transport function by looking at current-voltage (I-V) curves, which were generated using a RAMP protocol applied at the peak of the urate-induced currents (Fig. 3E). As published previously, we have found a quasi-linear I-V relationship from −120 to 60 mV^12^, indicating that the membrane voltage mainly acts as a driving force for the urate transport (Fig. 3E). The I-V curves for WT hSLC2A9 and Y298Q were similar, whereas N429H indicated a much lower urate transport activity. In contrast, uptake assays show the capacity to transport fructose was not affected by mutations Y298Q and N429H, which exhibited similar fructose transport capability to WT hSLC2A9, (Fig. 3F) (One-way ANOVA, *p < 0.05). Interestingly, for SLC2A9b, which typically binds fructose with high affinity, the N429H mutant has a four-fold lower affinity and a higher capacity for fructose transport with a two-fold increase in V_MAX_ (Suppl. Fig. 3).

### Replacement of cysteine residues in hSLC2A9 with equivalent residues from hSCL2A5 reveals role of cysteine residues in hSLC2A9

**Figure 4:**
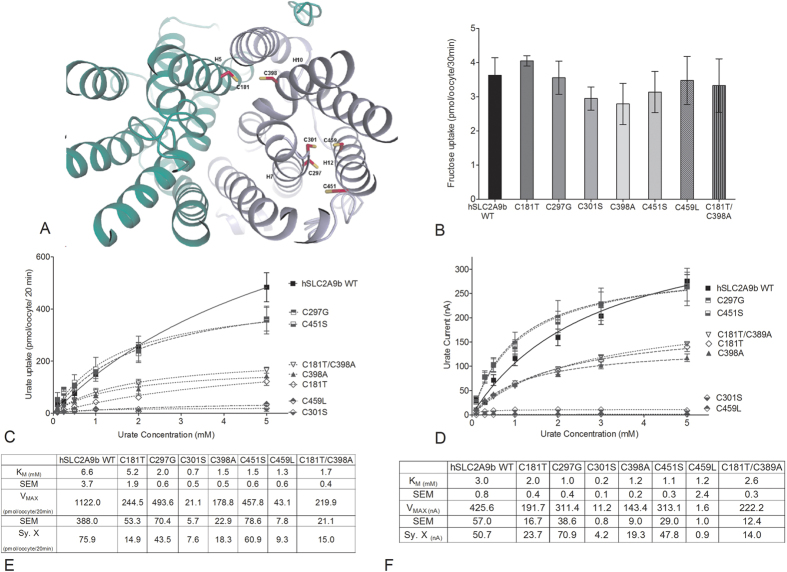
Urate and fructose transport mediated by WT hSLC2A9 and its cysteine mutants. Panel (A) Molecular model of human SLC2A9b with predicted locations of cysteine residues. View from the extracellular side of the outward facing conformation of hSLC2A9b. Panel (B) ^14^C fructose uptake mediated by WT hSLC2A9 and its cysteine mutants. Bar graphs represent fructose uptake activities, which were corrected for non-specific transport measured in control water injected oocytes from the same batch of oocytes (n ≥ 3, One-way ANOVA, *p < 0.05). Panel (C) Michaelis-Menten curves of ^14^C urate kinetics of hSLC2A9 WT (◼) and its mutants C297G (⬒), C451S (⬓), C181T (♢), C398A (▴), double mutant C181T/C398A (▿), C301S (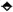), and C459L (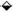).Uptake activity was corrected for non-specific transport measured in control water injected oocytes from the same batch of oocytes. Panel (**D**) Michaelis-Menten curves of urate-induced currents of WT hSLC2A9 and its cysteine mutants. Panel (E) ^14^C urate kinetic constants of the 3 isoforms (n ≥ 3). Panel (F) Urate-induced current kinetic constants of the WT and cysteine mutants (n ≥ 15 oocytes from 3 frogs).

The hSLC2A9b model predicted that cysteine residues C181 and C398 lie close to the aqueous pore of the transporter, (Fig. 4A). C297 and C301 in H7 are facing away from the aqueous pore; however, these two cysteines are facing another two cysteine residues, C451 and C459 in H12. In particular, this outward facing model predicted that C301 and C459 are very close to each other with a predicted distance of 3.5 angstroms (Fig. 4A). To assess the role of these cysteine residues in the specific urate transport capacity of hSLC2A9b we mutated these hSLC2A9b cysteine residues into the corresponding residues found in hSLC2A5. Then, we tested their functional roles using radiolabelled urate flux measurements, TEVC, and exposed these mutants to an oxidizing agent, p-chloromercuribenzene sulphonic acid (pCMBS). Fructose transport assays were used as internal controls for protein integrity and functionality. As shown in Fig. 4B, all mutants transported fructose similarly as WT hSLC2A9b (One-way ANOVA, *p < 0.05), indicating that these cysteine residues do not play a role in hexose transport.

Our ^14^C urate flux studies demonstrate that both WT hSLC2A9b and its cysteine mutants exhibited Michaelis-Menten type kinetics for urate transport (Fig. 4C). However, urate transport activities varied among the cysteine mutants (Fig. 4C and E). Urate kinetics of C297G and C451S were similar to WT, whereas higher urate affinities and lower urate transport capacities (lower K_M_ and V_max_ values) were seen in C181T, C398A, and the double mutant C181T/C398A. On the other hand, urate transport activities were dramatically reduced in C301S and C459L, with their V_max_ values reduced by 50 and 20 fold, respectively, compared to WT. Their K_M_ values were also 10- and 5-fold lower than WT, respectively, indicating that both mutants have very high affinities for urate binding compared to WT (Fig. 4C and E). Moreover, our fructose kinetic analysis showed that C181T has a lower fructose transport V_MAX_ compared to WT hSLC2A9b (Suppl. Fig. 3).

Since urate transport mediated by hSLC2A9 is an electrogenic process^12^,^18^,^19^, a TEVC method was also used to examine urate transport by hSLC2A9 and its cysteine mutants. Single oocytes were clamped at a holding potential of −30 mV, then superfused with different concentrations of urate followed by one-minute wash with standard transport media (STM). Induced outward currents were collected at the peak of each urate-induced current and averaged. As shown in Fig. 4D and F, urate kinetics obtained from TEVC and flux studies were similar. Urate transport kinetics were also similar among WT hSLC2A9b, C297G and C451S. In contrast, higher urate affinity and lower urate transport capacity (lower K_M_ and V_max_ values) were seen in C181T, C398A, and the double mutant C181T/C398A. Interestingly, urate-induced currents were dramatically reduced in C301S and C459L. Shown in Fig. 4F, C301S had a 10 times lower K_M_ and 50 times lower V_max_, whereas C459L had similar K_M_ but very low V_max_ (400 times lower) compared to WT. Also, C301S has very high urate affinity compared to WT. For all mutations, oocyte expression analysis indicted normal expression comparable to WT expression (Suppl. Fig. 2). Furthermore, our fructose kinetics analysis showed that C301S had a higher fructose transport V_MAX_, whereas C181T had a lower fructose V_MAX_ compared to WT hSLC2A9b (Suppl. Fig. 3) further showing these mutations did not disrupt the native structure. These results suggest that both C301S and C459L mutants of hSLC2A9b have a very low urate transporting capacity.

**Figure 5:**
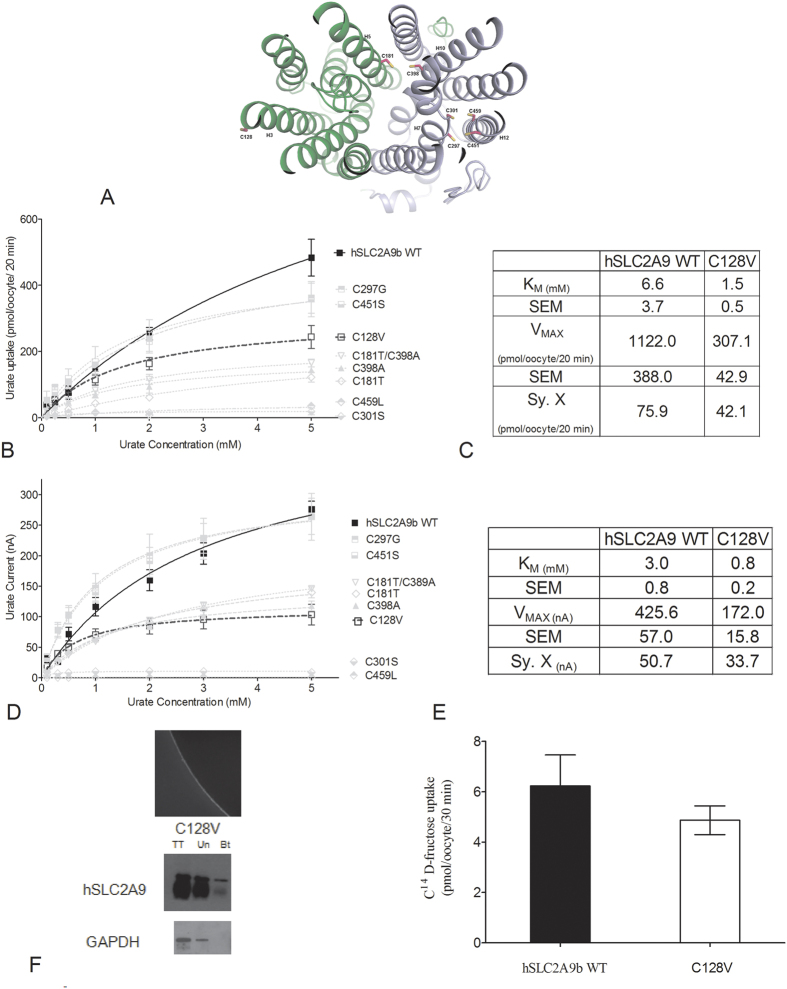
Urate and fructose transport mediated by WT hSLC2A9 and its C128V mutant. Panel (A) Michaelis-Menten curves of ^14^C urate kinetics of hSLC2A9b WT (◼) and C128V (◻).Panel (B) ^14^C urate kinetic constants and the standard error of the regression (Sy. X) of the 2 isoforms (n = 3). Panel (C) Michaelis-Menten curves of urate-induced currents of WT hSLC2A9b WT and C128V mutant. Panel (D) Urate-induced current kinetic constants and the standard error of the regression (Sy. X) of the WT and C128V mutant (n = 15 oocytes from 3 frogs). Panel (E) Representative pictures of immunohistochemistry and Western blot analysis of protein expression of C128V mutant expressing oocytes. Panel (F) ^14^C fructose uptake mediated by hSLC2A9b WT and C128V mutant (n = 3).

Residue C128 was not predicted by the hSLC2A9b model to sit in the central area of the transmembrane helices, as the other cysteine residues, but is in H3 facing the intra-membrane space (Fig. 5A). We postulated that this cysteine is also important for transport function that it may form a disulfide bridge between two hSLC2A9 monomers to form a dimer. We substituted C128 by valine, a corresponding residue in hSLC2A5 to examine its urate transport function. C128V in hSLC2A9 caused loss in urate transport capacity and increased urate binding affinity. Shown in Fig. 5B–D, both V_MAX_ and K_M_ of C128V were smaller compared to WT transporter. The urate transport kinetics curve of C128V was similar to those of C181T, C398A, and the double mutant C181T/C398A. In addition, C128V transported fructose similarly to WT hSLC2A9b (Fig. 5G). Data from our immunohistochemistry and biotinylation experiments indicated that C128V expresses on the oocyte membrane to a similar extent as the WT protein (Fig. 5F).

**Figure 6:**
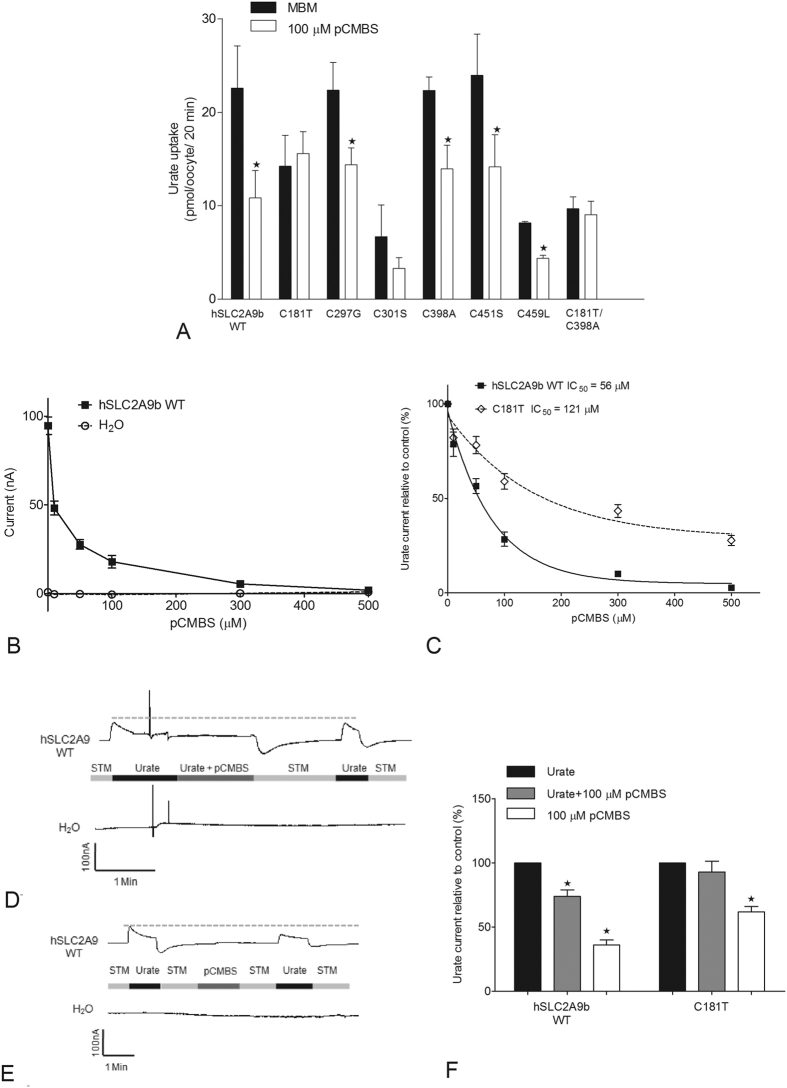
pCMBS inhibition experiments. Panel (A) pCMBS screening in ^14^C urate uptake mediated by WT hSLC2A9 and its cysteine mutants. Bar graphs represent 100 μM urate uptake activities before (dark) and after (white) 100 μM pCMBS treatments. Data was corrected for non-specific transport measured in control water injected oocytes from the same batch of oocytes (n ≥ 3, unpaired t-test, *p < 0.05). Panel (B) pCMBS inhibition curves of urate-induced currents of WT hSLC2A9 (◼) expressing and control water injected (○) oocytes (n ≥ 15 oocytes from 3 frogs). Panel (C) pCMBS inhibition curves of urate-induced current of WT hSLC2A9 (◼) and C181T (◊) protein expressing oocytes. Data were corrected with basal currents before the pCMBS treatment. IC_50_ is the pCMBS concentration for 50% inhibition of the urate-induced current (n ≥ 15 oocytes from 3 frogs). Panel (D and E) Representative trace of urate protecting pCMBS inhibition and control experiment, respectively. Urate-induced current was elicited by perfusing an oocyte expression WT hSLC2A9 (upper trace) or water injected oocyte (lower trace) with 1 mM urate (first urate-induced peak) followed by 1 min 100 μM pCMBS incubation. The oocyte then was washed with STM for at least 1 min to remove both extracellular pCMBS and urate. Finally, the oocyte was perfused with 1 mM urate again (second urate-induced peak). Panel (F) Urate protecting pCMBS inhibition experiments of WT hSLC2A9 and C181T. Bar graphs are data corrected to control (first peak of urate-induced current before pCMBS treatment) currents (dark) currents after oocyte in both 1 mM urate and 100 μM pCMBS (grey), and currents after oocyte in only 100 μM pCMBS (white) (n ≥ 15 oocytes from 3 frogs, One-way ANOVA, *p < 0.05).

Additionally, we utilized pCMBS to examine which cysteine residue(s) is (are) located within the substrate transporting aqueous pore in hSLC2A9. We screened for inhibition of urate transport by pCMBS on all of the cysteine mutants of hSLC2A9 with both flux and TEVC studies Fig. 6A. Only the C181T and the double mutant C181T/C398A showed protection from pCMBS inhibition; all other cysteine mutants showed similar inhibition to WT hSLC2A9b (unpaired t-test, *p < 0.05) in a dose dependent manner, Fig. 6B and C. The urate-induced current in WT hSLC2A9b was abolished completely after incubation with 500 μM pCMBS with an IC_50_ of 52.5 μM. In contrast, the IC_50_ for C181T was 110.2 μM, which indicates that it requires twice the concentration of pCMBS to inhibit 50% of the urate-induced current. Two representative traces shown in Fig. 6D and E are from single hSLC2A9b protein expressing (upper) and water injected (lower) oocytes clamped at −30 mV. Both protein expressing oocytes and the water injected control oocytes were perfused with 1 mM urate followed by 1 min 100 μM pCMBS incubation in the presence (Fig. 6D) or absence (Fig. 6E) of urate. These results illustrate that urate-induced currents reduced significantly after pCMBS was applied to the oocyte. In addition, experimental data in Fig. 6F showed that urate-induced currents have a higher percentage of recovery when the oocyte was treated with both urate and pCMBS compared to the one with only pCMBS. This phenomenon is true for both WT hSLC2A9b and the C181T mutant (One-way ANOVA, *p < 0.05). Consequently, these experiments confirm that urate can protect the protein from the pCMBS inhibition and that the substrate binding site may be close to the relevant cysteine residues.

### Role of H7 in urate/fructose transport in chimæric protein hSLC2A9_(7)5_

**Figure 7:**
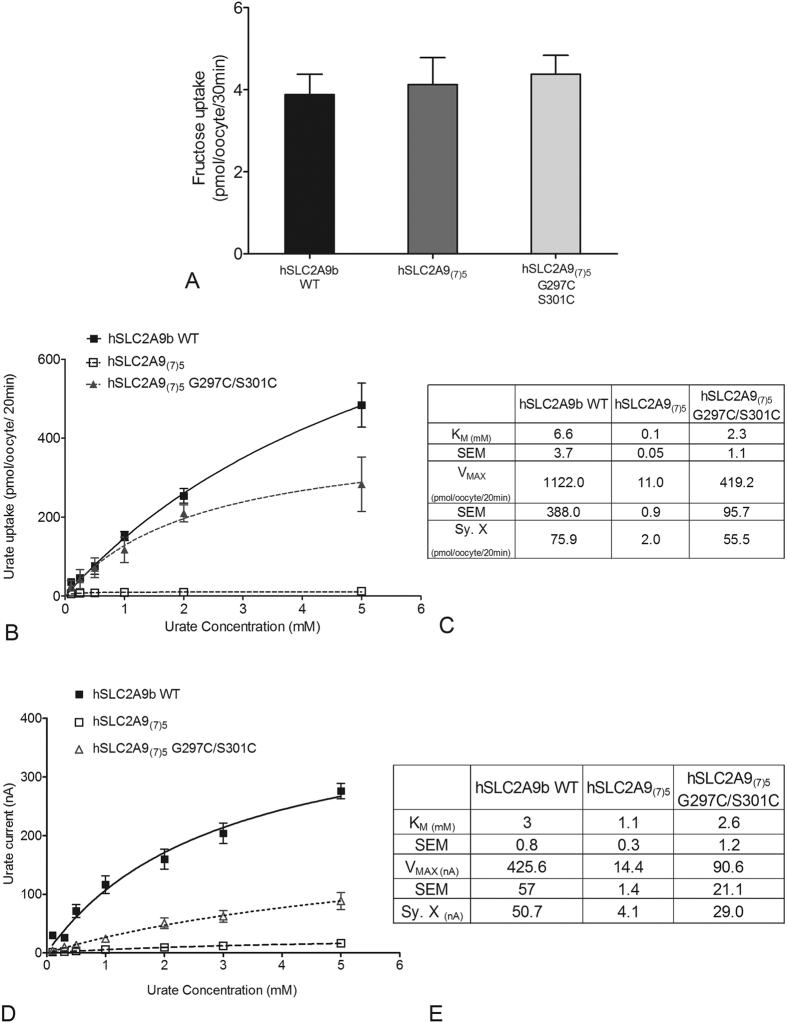
Fructose and urate transport mediated by WT hSLC2A9, its chimæra hSLC2A9_(7)5_ and hSLC2A9_(7)5_ G297C/S301C. Panel (A) ^14^C fructose uptake mediated by WT hSLC2A9 and its chimæric mutants. (n ≥ 3, One-way ANOVA, *p < 0.05). Panel (B) Michaelis-Menten curves of ^14^C urate kinetics of hSLC2A9 WT (◼), its chimærichSLC2A9_(7)5_ (◻), and hSLC2A9_(7)5_ G297C/S301C (▴). Panel (C) ^14^C urate kinetic constants and the standard error of the regression (Sy. X) of the 3 isoforms (n ≥ 3). Panel (D) Michaelis-Menten curves of urate-induced currents of WT hSLC2A9 and its chimæric mutants. Currents were measured by TEVC. Panel E. Urate-induced current kinetic constants and the standard error of the regression (Sy. X) of the WT and its chimæric mutants (n ≥ 15 oocytes from 3 frogs).

Within the SLC2A protein family, H7 has been implicated in forming part of the translocation pore and playing an important role in substrate binding. Our previous publications have suggested that an isoleucine in H7 at the exofacial surface of Class II hSLC2As is a main determinant of substrate selection^10^,^11^,^12^. In this study, we further identified a cysteine residue, C301 that is essential for urate transport in hSLC2A9. Therefore, we continue to search for other possible sites that might be important in substrate transport in hSLC2A9, and we predicted that L303 would be another important residue in this glucose transporter. We used site-directed mutagenesis to generate a hSLC2A9 L303V (valine is a corresponding residue in hSCL2A5) mutant. Results from both ^14^C fructose kinetics and TEVC urate kinetics indicated that substitution of leucine by valine affects fructose transport but not urate transport (Suppl. Figs 2 and 3). Affinity and capacity of the fructose transport increased in L303V compared to WT hSLC2A9b (Suppl. Fig. 3). This data suggested that I303 in hSLC2A9b is also a critical residue that forms a hydrophobic network with other hydrophobic residues on the other transmembrane helices, which regulates the rigid body of the protein structure and affects the protein substrate selectivity. We know many residues have been identified in transmembrane helix 7 (H7) of glucose transporters as important for substrate binding of hexoses or their translocation. Aside from our previous study showing the importance of H7 residues on substrate selection^10^,^12^ the H7 in hSLC2A9, as a whole, has not previously been examined for its role in substrate transport. Therefore, we further analyzed the function of H7 in distinct substrate recognition between fructose and urate by creating a chimæra called hSLC2A9_(7)5_ in which the H7 of hSLC2A5 protein was used to replace the H7 of hSLC2A9b. We measured both the fructose (as an internal control) and urate fluxes in the chimæra. ^14^C fructose flux experiments were performed as a control to verify that the chimæra is functional (Fig. 7A). hSLC2A9_(7)5_ and WT transporter exhibited similar fructose transport activities (Fig. 7A, One-way ANOVA, *p < 0.05). Additional fructose kinetics analysis demonstrated that hSLC2A9_(7)5_ actually has a higher fructose transport capacity, V_MAX_, compared to WT hSLC2A9b (Suppl. Fig. 3).

In contrast, ^14^C urate fluxes (Fig. 7B and C) and TEVC measurements (Fig. 7D and E) showed that the hSLC2A9_(7)5_ chimæric protein loses its urate transport ability almost completely, with very low affinity and capacity compared to WT hSLC2A9. These results allowed us to conclude that the chimæric protein hSLC2A9_(7)5_ has a very limited ability to transport urate compared to WT, demonstrating the importance of H7 in urate transport.

**Figure 8:**
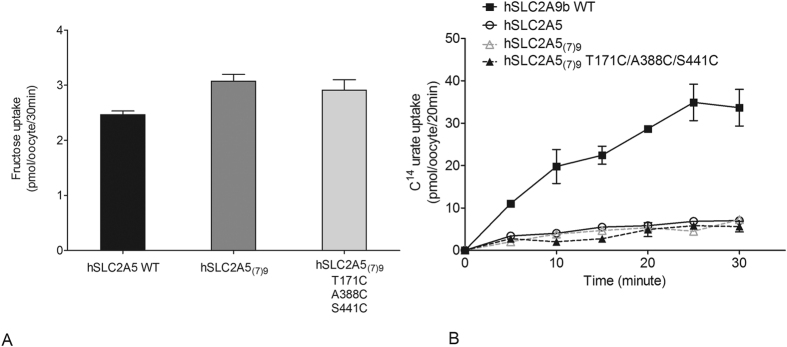
Fructose and urate transport mediated by WT hSLC2A5, its chimæra hSLC2A5(7)9 and hSLC2A5(7)9 T171C/A388C/S441C. Panel (A) ^14^C fructose uptake mediated by WT hSLC2A5 and its chimæric mutants. (n ≥ 3, One-way ANOVA, *p < 0.05). Panel (B) ^14^C urate uptake time course experiment of hSLC2A9, WT (◼), WT hSLC2A5 (⚪), hSLC2A5_(7)9_ (▵) and hSLC2A5_(7)9_ T171C/A388C/S441C (▴).

An additional approach to determine the role of H7 in urate transport was to substitute H7 of hSLC2A5 with that from hSLC2A9, generating a chimæra called hSLC2A5_(7)9_.^14^C fructose flux studies again showed that the chimæra retains expression and functionality (Fig. 8A) (One-way ANOVA, *p < 0.05). However, the time course of urate uptake mediated by hSLC2A5 and SLC2A5_(7)9_ demonstrated no significant gain of urate transport function (Fig. 8B). Consequently, it appears that the H7 alone is insufficient to convert hSLC2A5 into a urate transporter.

Furthermore, we mutated two cysteine residues G297C and S301C back into the H7 of the hSLC2A9_(7)5_ chimæra to ascertain whether these two cysteines would reinstate urate transport function. Again ^14^C fructose flux assays were used as an internal control to ensure that hSLC2A9_(7)5_ G297C/S301C is a functional protein (Fig. 7A) (One-way ANOVA, *p < 0.05). Both the ^14^C urate flux measurements (Fig. 7B and C) and TEVC data (Fig. 7D and E) showed that urate transport was partially recovered when the two cysteine residues were reintroduced into H7. This indicates that while these two cysteine residues in H7 are essential they are not sufficient for full urate transport activity. In addition, we introduced the three key cysteines from hSLC2A9b into the chimæric hSLC2A5_(7)9_ as hSLC2A5_(7)9_-T171C/A388C/S441C. Although this cysteine containing hSLC2A5_(7)9_ transports fructose as well as WT hSLC2A5, it showed no significant gain of urate transport function (Fig. 8B). Consequently, this result demonstrates that these important corresponding cysteine residues from hSLC2A9, were insufficient to induce urate transport activity in hSLC2A5_(7)9_.

## Discussion

Recent crystal structures of glucose transporter 1 and 5 (SLC2A1 and SLC2A5) postulated an alternating access mechanism as a likely model for the translocation of their sugar substrates. Both structures revealed that this transport mechanism involves substrate recognition/binding residues within a central binding pocket, the movement of the intra-cellular helices domains, and the movement of transmembrane helices domains. On the other hand, a docking study of xylose transporter (XlyE) proposed a gated sugar channel with sequential binding sites within the transporter aqueous pore to help sugar across the transporter through diffusion^20^. This current report further extends our understanding of the mechanism for not only sugar but also urate transport in a glucose transporter by examining the substrate-binding pocket and the role of the transmembrane H7 in hSLC2A9.

From the hSLC2A9 molecular model, we predict both fructose and urate bind within the same pocket as proposed for the SLC2A5 crystal structure, albeit with slightly different orientations and side chain contacts. This difference may help to explain why we had difficulty in showing competition between urate and fructose for transport^19^, although at least one report did find an effect at high concentrations^21^. Our docking study and functional data indicated that only N429 is related specifically to urate transport mediated by hSLC2A9, in contrast, the Y298Q mutation has no effect on urate transport. Together with our urate docking and kinetic studies, we postulate that N429 is one of the key residues for holding urate in the perpendicular position within the binding pocket. Another hSLC2A9 structural model based on XylE suggested that another asparagine, N433 (N462 in full length hSLC2A9), as the possible binding site for urate^22^. This N433 is very close to the one (N429) we predicted with our model. This further emphasizes that asparagine is important for urate transport in hSLC2A9. However, additional experiments are needed to determine whether both N429 and N433 are equally important or only one is essential.

Functional studies of cysteine residues replacements with the corresponding residues from hSLC2A5 show that C297 and C451 are not required for urate transport in hSLC2A9, conversely, C181T, C398A and the double mutants C181T/C398A reduced urate transport activity significantly. These data suggest that C181 and C398 are functionally important. Furthermore, C181 was the only cysteine found to be responsible for the pCMBS inhibition effect, and the protein became insensitive to pCMBS when we mutated this C181 into threonine. This result suggests that C181 has a SH- group facing the aqueous environment of the transport pore, which can react with the thiol-group-reactive reagent. This supports our homology model of hSLC2A9 (Figs 4A and 5A) which also indicates C181 faces the translocation pore. These findings are comparable with other transporters, like the equilibrative nucleoside transporters (ENTs) and concentrative nucleoside transporter (CNT) proteins^23^,^24^,^25^. For instance, a single cysteine, C140, was located within the substrate translocation channel in rat ENT2, where it reacted with pCMBS^23^. Moreover, cysteines in human ENT1 were reported responsible for inhibitor binding and substrate transport function. Although our computer model also predicted that C398 locates within the aqueous surroundings, functional study supported that its SH- group is not facing the aqueous pore; instead, the SH-group of C398 is facing away from the pore towards other side chains (Fig. 5A).

Interestingly, both the C301S and C459L mutants of hSLC2A9 lost their urate transport ability. Cysteines are well known to maintain protein structures and conformation by forming disulfide bridges^26^,^27^,^28^ between transmembrane helices. Our hSLC2A9 model indicates that these two cysteines found in H7 and H12 are in close proximity (3.5 Angstroms), suggesting a disulfide interaction could form between them during the translocation process. Thus, this interaction would be lost when one of these two cysteines is mutated and thus changing the TMs’ orientation and the protein’s overall conformation. Another possible explanation is that the difulside interaction helps to bring other residues close to each other, such as the hydrophobic residues, within the membrane to tightly hold the helices of the protein in a proper conformation again affecting function^22^,^26^,^29^. In parallel with this assumption, our previous study pointed out that a hydrophobic residue, isoleucine 335 in H7 in hSLC2A9, formed hydrophobic interactions with other hydrophobic residues on the other side of the TMs, resulting in a more rigid protein body; again allowing the protein to translocate both urate and fructose^12^.

Moreover, we speculate that C128 is a site that might form a disulfide bridge between two hSLC2A9 monomers that allows them to form a dimer during transport. Hebert and Carruthers^30^ reported the dimerization in hSLC2A1 through a disulfide bond to stabilized the transporter structure, thus enhance the hexose transport function. Zottola *et al*.^29^ illustrated two cysteines, C347 and C421 on the C-terminal half are critical for intramolecular disulfide formation for tetramerization. Substitution of these two cysteines, C347 or C421 to serine significantly reduces tetrameric GLUT1-specific epitopes. Therefore, it is very possible that the thiol group of residue C128 forms an intramolecular disulfide linkage between the two hSLC2A9 monomers similar as the ones in hSLC2A1, thus enhancing the urate transport activity. When we break the linkage by changing the cysteine into valine, the thiol groups were no longer present, hence altering the structure of the hSLC2A9 and the urate transport ability. However, future studies with purified hSLC2A9 protein are needed to confirm the formation of the disulfide bridges between cysteine residues, C301 and C459, and the dimerization hypothesis in hSLC2A9.

To probe the role of H7 in urate transport mediated by hSLC2A9 we constructed two chimæric proteins, hSLC2A9_(7)5_ and hSLC2A5_(7)9_. Replacement of H7 inhSLC2A9 with that from hSLC2A5 cause a substantial loss of urate transport without affecting fructose transport in the chimæric protein hSLC2A9_(7)5_. This suggests that elements within H7 are critical for urate transport, however, substitution of H7 from hSLC2A9 into hSLC2A5 did not induce urate transport. This indicates that H7 alone is not sufficient to bring urate transport into other glucose transporters and that residues contributing to the full binding pocket must come from other TMs. With the substitution of cysteine residues back into the H7 in chimæric protein, the hSLC2A9_(7)5_ was able to recover partial but not full urate transport. This provides further support that the cysteines in H7 are essential for the protein to transport urate. However, other residues are also important for full recovery of the transport activity. When we replaced the residues T171/A388/S441, in the hSLC2A5_(7)9_ protein with the corresponding cysteines from hSLC2A9, the chimæric protein still did not gain urate transport function. These findings allow us to conclude that both H7 and its cysteine residues are critical for urate transport in hSLC2A9; yet, they are not alone sufficient to induce urate transport in hSLC2A5, and other helices and residues are also crucial for urate binding and translocation. It is also noted that mutants like hSLC2A9_(7)5_, C301S, L303V, and N429H have higher fructose transport capacities compared to WT suggesting that these mutants, with the fructose transporter hSLC2A5 helices and residues, can handle fructose much better than hSLC2A9 WT.

As mentioned above, N429 and possibly N433 are crucial for urate binding. Thus, with the absence of the two residues, urate might not be properly associated with other residues within the binding pocket. Moreover, the SLC2A5 crystal indicates that H10 is also critical in substrate recognition, binding and translocation^7^. Therefore, it would be very interesting to see whether putting N429, N433, H7 and H10 from hSLC2A9 into hSLC2A5 would help the chimæric hSLC2A5 gain urate transport.

In summary, hSLC2A9 is a very special glucose transporter in that it can transport not only glucose and fructose but also an organic anion, urate. This makes hSLC2A9 a potential target for pharmaceutical agents for treating diseases like diabetes or gout. This study illustrates that three cysteine residues (C181, C301 and C459) are critical for urate transport and that N429 in hSLC2A9 may form part of the urate binding pocket. The inability to induce urate transport in hSLC2A5 with both H7 and the cysteine residues from hSLC2A9 leads us to a question: What is/are other TM(s) or residue(s) might also be essential for urate transport by hSLC2A9 ? In addition, we observed that, from multiple sequence alignments (Suppl. Fig. 1), the Class II glucose transporters have many cysteines presenting in their TMs. For example, hSLC2A11 has 16 cysteines and 12 of them are within its TMs. Therefore, it will be very interesting to further explore what are the actual physiological substrates mediated by these transporters, and what are the roles of the cysteines involved in their transport processes.

## Experimental Procedures

### hSCL2A9 modeling and molecular docking

The structure of the human glucose transporter protein (hSLC2A9) was obtained by submitting the protein sequence for automated protein structure modeling using the I-TASSER pipeline^31^,^32^. This method aided in construction of a model with open/outward facing conformation using multiple threading alignments. The open conformation crystal structure of rat SLC2A5 (PDB:4YBQ) was used as a template^7^. The C-score for the model was 0.49 suggesting the generation of reliable models for further computational studies. The structure thus obtained was used to perform blind molecular docking to capture the potential ligand-binding poses utilizing the program AutoDockVina (v 1.0.2)^33^. This program docks a flexible ligand against the protein using a sophisticated gradient optimization method. Hence, this method was shown to be more accurate compared to previous versions of AutoDock^34^,^35^. The ligand structures were drawn using MarvinSketch 5.3.1 (ChemAxon). The UCSF Chimera program (http://www.cgl.ucsf.edu/chimera) was used to prepare the ligand structures for input to AutoDockTools (ADT) version 1.5.4 by adding Gasteiger charges (computed using ANTECHAMBER) and running 10,000 steps of energy minimization^36^. ADT was used to add polar hydrogens to both protein and ligand structures. The structures were then saved as PDBQT files for input into AutoDockVina (v 1.0.2) (http://vina.scripps.edu). The parameter “Exhaustiveness” which determines how comprehensively the program searches for the lowest energy conformer, was set to default value (eight). To validate the blind docking technique, the docking was repeated by decreasing the search space. Docking was performed independently for fructose and urate as ligands.

### Plasmid construction

We received the original human wild type (WT) hSLC2A9 plasmid as a gift from Kelle Moley (School of Medicine, Washington University, USA), and we constructed it into pGEM-HE vector for oocyte expression. Site-directed mutagenesis was performed using the QuikChange II site-directed mutagenesis kit (Stratagene) to generate the point mutations (C181T, C297G, C301S, C398A, C451S, C459L, double/triple mutations C181T/C398A, G297C/S301C of hSLC29_(7)5_, and T171C/A388C/S441C of hSLC2A5_(7)9_, and chimæra mutation hSLC2A9_(7)5_ of the wild type hSLC2A9 and hSLC2A5_(7)9_ of hSLC2A5. Fast cloning protocol was used in chimæra protein primer design^37^. All primers are shown in Suppl. Table 1, 2. and 3. DNA plasmids were sequenced by Macrogen (Maryland, USA) to ensure accuracy of the construction.

### mRNA preparation & *Xenopus laevis* oocyte micro injection

mRNA preparation and microinjection were performed as previously described^12^,^18^. In short, wild type and mutant plasmids were linearized with *NheI* and transcribed *in vitro* with T7 polymerase mMESSAGE mMACHINETM (Ambion). *Xenopus laevis* oocytes were treated with collagenase and defoliculated before injection^18^. Oocytes were injected with 20 ng mutant plasmid mRNA and incubated in modified Barth’s medium (MBM, 88 mM NaCl, 1 mM KCl, 0.33 mM Ca(NO_3_)_2_, 0.41 mM CaCl_2_, 0.82 mM MgSO_4_, 2.4 mM NaHCO_3_, 10 mM Hepes, 2.5 mM sodium pyruvate, 0.1 mg/ml penicillin and 0.05 mg/ml gentamycin sulfate, pH 7.5) for 4 to 5 days at 16–18 °C prior to functional assays. All chemicals were obtained from Sigma-Aldrich (Oakville, Ontario, Canada) unless otherwise stated. Same amount of water was injected into oocytes (control) for control experiments. NanoDrop 1000 Spectrophotometer V3.7 (Thermo Fisher Scientific, USA) was used to determine the concentration of mRNA of all isoforms.

Xenopus were housed, anesthetized and euthanized following protocols approved and authorized by University of Alberta Animal Research Ethics Committee.

### Radiotracer flux

Radiotracer flux studies were conducted at room temperature (RT) at 20–22 °C. ^14^C labeled urate (8-^14^C urate) (Moravek) and ^14^C fructose [^14^C-(U)] was used in flux studies. As previously described^12^,^19^, urate transport was measured by incubating oocytes with 200 μL urate solution ranging from (50 μM to 5 mM). Oocytes were incubated for 20 min, which was within the linear range of urate uptake. Transport reaction was stopped by washed with ice cold MBM; individual oocytes were placed into scintillation vial for radioactivity measurements by liquid scintillation counting. All radioactivities were measured using a Beckman LS6500 liquid scintillation counter (Fullerton, CA, USA).

#### Two-microelectrode voltage clamp

Electrophysiology experiments were carried out by TEVC technique with GeneClamp 500B (Molecular Devices Inc. Sunnyvale, CA, USA)^12^. Sodium containing transport medium (STM, 100 mM NaCl, 2 mM KCl, 1 mM CaCl_2_, 1 mM MgCl_2_, 10 mM Hepes, pH 7.5 with Tris Base) was used to perfuse oocytes to obtain a current base line before adding experimental substrates. Urate-induced current (urate concentrations in the range from 0.05 mM to 5 mM in STM) at holding potential of −30 mV was obtained using GAP-Free protocol^12^. Data were expressed as urate-induced mean peak current. Current-voltage (I-V) curves were obtained using a RAMP protocol, in which voltage changed from −120 to 60 mV for a 3-second period. The RAMP protocol was applied at the peak current following addition of a substrate or inhibitor. Degidata 1320 A converter and pClamp8 (Axon Instruments, Union City, CA) were used for experiments and data analysis.

### p-Chloromercuribenzene sulfonic acid (pCMBS) treatments

^14^C urate flux was used to screen the inhibition effect of pCMBS on WT hSLC2A9 and its cysteine mutants. Experiments were performed as previously described with modifications^38^,^39^. Briefly, oocytes expressing WT or mutant hSLC2A9 were incubated in of 100 μM pCMBS, or MBM for control, for one minute followed by three times washes with RT MBM. Both pCMBS treated oocytes and control oocytes were subjected to the 20 min ^14^C urate flux assay to determine the amount of radioactivity oocytes had taken up.

Furthermore, we used the TEVC to reveal more properties of the pCMBS effect on WT hSLC2A9 and C181T. In the TEVC study, protein injected oocytes were perfused with 1 mM urate followed by 1 min STM wash; then pCMBS (range from 10 μM to 500 μM) was directly added to the oocyte holding chamber and incubate for 1 min. pCMBS was washed out for 1 min to ensure all the pCMBS was clear. Finally, oocytes were perfused with 1 mM urate again to obtain the urate-induced current. Peak values of the urate-induced currents, before and after pCMBS treatments, were collected for comparison and IC_50_ calculation, which IC_50_ is the concentration of pCMBS that inhibits 50% of the urate-induced currents. Additional experiments were performed to distinguish whether urate will protect the pCMBS inhibition effect. Experiments are as follows: oocytes were perfused with 1 mM urate, when the urate-induced current reached its plateau, 100 μM pCMBS in 1 mM urate were administrated directly to the oocyte holding chamber and incubated for 1 min. Both urate and pCMBS were washed away with STM after 1 min. Finally, oocytes were perfused again with 1 mM urate to obtain urate-induced current.

### Protein expression levels of hSLC2A9, hSLC2A5, and their mutants were determined by immunohistochemistry, biotinylation and Western blot (Suppl. Fig. 2)

Immunohistochemistry - was used to determine relative protein levels at the *X. laevis* oocyte membrane. Oocytes were washed with Phosphate Buffer Saline (PBS; 137 mM NaCl, 2.78 mM KCl, 4.3 mM Na_2_HPO_4_, 1.5 mM KH_2_PO_4_, pH 7.4), then fixed in 3% paraformaldehyde (PFA) in PBS for 15 minutes at RT. After fixation, oocytes were washed with 50 mM NH_4_Cl and permeabilized with 0.1% Triton X-100. Oocytes were then blocked with 2% *bovine serum albumin* (BSA) for 30 minutes followed by incubation with primary hSLC2A9 anitibody (Cedarlane, Canada) in blocking buffer for 1 hour at RT, and then secondary antibody, rabbit anti goat conjugated to Alexa 488 (Invitrogen, USA). Oocytes were mounted using Vectashield mounting medium (Vector Laboratories, Inc. Burlingame, CA USA) on slides with secure-seal spacers. Protein expression was determined by Wave FX confocal microscopy (Quorum Technologies, ON, Canada) of the fluorescent secondary antibody.

### Biotinylation

Protein expression was analysed using cell surface biotinylation followed by Western blot of each isoform based on a previously published protocol with modifications^12^. Oocytes were washed three times with PBS (pH 8.0); then, they were incubated in 2 mM Sulfo-NHS-LC-Biotin (Pierce) in PBS at room temperature for 30 minutes. The reaction was stopped by washing with quenching buffer (192 mM Glycine and 25 mM Tris-HCl in PBS, pH 7.5). Oocytes were lysed with RIPA buffer (150 mM NaCl, 1% Triton-X-100, 1% deoxycholic acid, 0.1% SDS, 1 mM EDTA, 10 mM Tris-HCl, pH 7.5). Lysates were incubated with streptavidin agarose (Pierce) at 4 °C overnight. Beads were centrifuged at 3000 rpm. Biotinylated proteins were resuspended using SDS sample buffer and subjected to SDS-PAGE. Proteins from SDS-PAGE were transferred to nitrocellulose membranes and blocked with 3% skim milk in PBST (0.05% Tween 20 in PBS). After blocking, the membrane was probed with primary hSLC2A9 antibody (Cedarlane, Canada) and anti-rabbit secondary antibody (Abcam, Cambridge, MA, USA).

### Data analysis

Protein amino acid sequence alignments were performed using Clustal OMEGA (http://www.ebi.ac.uk/Tools/msa/clustalo/). Graphpad 5.0 was used to analyze all the data. Urate kinetic data were fitted using non-linear regression. Current-voltage (I-V) curves were graphed using X-Y plots. Fructose flux studies, pCMBS inhibition experiment and protein expression data were analyzed by One-way ANOVA for the flux studies and Student unpaired t-test in TEVC studies. Values were considered significant when p < 0.05. Cell surface biotinylation of protein was detected by Western blot and the protein band intensity was analyzed by ImageJ. Data were expressed as percentage of biotinylated protein to the total expressing protein designated as 100%.

## Additional Information

**How to cite this article**: Long, W. *et al*. Identification of Key Residues for Urate Specific Transport in Human Glucose Transporter 9 (hSLC2A9). *Sci. Rep.* **7**, 41167; doi: 10.1038/srep41167 (2017).

**Publisher's note:** Springer Nature remains neutral with regard to jurisdictional claims in published maps and institutional affiliations.

## Supplementary Material

Supplementary Information
