# Supplementary material for: Identification of Key Residues for Urate Specific Transport in Human Glucose Transporter 9 (hSLC2A9)
Source: Sci Rep. 2017 Jan 24;7:41167. doi: 10.1038/srep41167 (PMC5259734; doi:10.1038/srep41167)
Supplement: Supplementary Information [file srep41167-s1.doc]

**Identification of Key Residues for Urate Specific Transport in Human Glucose Transporter 9 (hSLC2A9)**

**Wentong Long^1^, Rashmi Panigrahi^2^,** **Pankaj Panwar^2^, Kenneth Wong^1^, Debbie O'Neill^1^, Xing-Zhen Chen^1^, M. Joanne Lemieux^2^, Chris I. Cheeseman^1^**

Supplementary Figure Legends, Figures & Tables.

Supplementary Figure 1. Multi-amino acid sequences alignments of Class I and II glucose transporter family proteins. Transmembrane helices (Hs) are highlighted in yellow color based the new GLUT1 crystal structure. Cysteine residues are highlighted in red color. Protein amino acid sequences alignments were performed by Clustal OMEGA (<http://www.ebi.ac.uk/Tools/msa/clustalo/>).

Supplementary Figure 2. Qualitative and quantitative determination of WT and mutant hSLC2A9 protein expression. Panel A. Representative pictures of immunohistochemistry and Western blot analysis of protein expression of water injected, WT hSLC2A9 and its cysteine mutants expressing oocytes. Tt: total protein; Un: Unbound protein; Bt: biotinylated protein. Panel B. Representative pictures of immunohistochemistry and Western blot analysis of protein expression of Y298Q, N429H and chimærahSLC2A9_(7)5_, and hSLC2A9_(7)5_ G297C/S301C mutants expressing oocytes. Panel C. Representative pictures of immunohistochemistry and Western blot analysis of protein expression of water injected, WT hSLC2A5, chimæric protein hSLC2A5_(7)9_ and hSLC2A5_(7)9_ T171C/A388C/S441C expressing oocytes. Total (black), Unbound (grey), and biotinylated (white) proteins of all isoforms. Panel D. Quantitative analysis of protein expression of all hSLC2A9 isoforms. Data were calculated from band intensities obtained from Image J and use a formula: % Biotinylated protein = (Total protein - Unbound protein)/Total protein x 100%. Protein expression levels shown as bar graphs with arbitrary units. (n≥3, One-way ANOVA, * p<0.05).

Supplementary Figure 3. **Panel A.** Michaelis-Menten curves of ^14^C fructose kinetics of hSLC2A9 WT (■), hALC2A9(7)5 (□), N429H (Δ), C181T (▼), C301S (▲), and L303V (◊). Fructose uptake was measured by incubating protein expressing oocytes in 200 µL fructose solution ranged from 100 µM to 5 mM for 30 minutes. **Panel B.** ^14^C Fructose kinetic constants and the standard error of the regression (Sy. X) of the six isoforms (n ≥3). **Panel C.** Michaelis-Menten curves of urate-induced currents of WT hSLC2A9 and its L303V mutant. **Panel D.** Urate-induced current kinetic constants of the WT and cysteine mutants (n ≥15 oocytes from 3 frogs).

Supplementary Table 1. Primers of cysteine, L303V, Y298Q, and N429H mutants of hSLC2A9.

Supplementary Table 2. Primers of chimæra protein construction, and primers of cysteine mutation in H7 of hSLC2A9_(7)5_.

Supplementary Table 3. Primers of chimæra protein construction of WT hSLC2A5, and cysteine mutation in hSLC2A5_(7)9_.

Supplementary Figure 1.

Supplementary Figure 2.

Supplementary Figure 3.

Supplementary Table 1.

Supplementary Table 2.

Supplementary Table 3.
